# Smoking cessation sharply reduced lung cancer mortality in a historical cohort of 3185 Chinese silicotic workers from 1981 to 2014

**DOI:** 10.1038/s41416-018-0292-6

**Published:** 2018-11-13

**Authors:** Lap Ah Tse, Xiaona Lin, Wentao Li, Hong Qiu, Chi Kuen Chan, Feng Wang, Ignatius Tak-sun Yu, Chi Chiu Leung

**Affiliations:** 1 JC School of Public Health and Primary Care, The Chinese University of Hong Kong, Hong Kong SAR, China; 2 School of Medicine, South China University of Technology, Guangzhou, China; 3 School of Public Health, The University of Hong Kong, Hong Kong SAR, China; 4 Pneumoconiosis Clinic, Tuberculosis and Chest Service, Department of Health, Hong Kong SAR, China; 5 Stanley Ho Centre for Emerging Infectious Diseases, the Chinese University of Hong Hong, Hong Kong SAR, China

**Keywords:** Occupational health, Epidemiology

## Abstract

**Background:**

Population-based studies showed an over 50% decrease in lung cancer risk after quitting smoking for 5–6 years, but the beneficial effect in silicotics remains unknown. We aimed to rectify this knowledge gap using a large historical cohort of 3185 Chinese silicotics since 1981 and followed-up till 2014.

**Methods:**

Baseline information on workers’ socio-demographics, smoking habits, occupational history, and medical history was collected. Smoking status was reassessed during follow-up. Multiple Cox proportional hazards model was performed to evaluate the impact of smoking cessation on lung cancer mortality.

**Results:**

Overall, 1942 deaths occurred and 188 lung cancer deaths were identified. Compared with never quitters, silicotics who were new quitters had almost halved their lung cancer risk [hazard ratio (HR) = 0.51, 95%CI: 0.34–0.76], while persistent quitters had a 53% risk reduction (HR = 0.47, 95%CI: 0.33–0.66). Lung cancer mortality approximately halved after quitting smoking for 10 years. While the risk kept decreasing with years since cessation, it did not reverse back to that of never smokers. Persistent quitters with small opacities tended to have higher beneficial effects than those with large opacities.

**Conclusions:**

Smoking cessation for 10 years halved lung cancer mortality among silicotics, while the beneficial effect was prominent for patients with small opacities.

## Introduction

Lung cancer remains the most common cancer with respect to both incidence and mortality worldwide, including Hong Kong.^1,2^ Silicosis is among the most important occupational diseases in Hong Kong,^3^ resulting from prolonged exposure to dust containing respirable crystalline silica during various types of work, including quarrying, tunneling, mining, and sandblasting.^4^ Silicosis and/or inhalation of respirable crystalline silica dust is evident to be the major occupational risk factor for lung cancer concluded by the International Agency for Research on Cancer.^5^ Silica along with asbestos, radon, heavy metals, and polycyclic aromatic hydrocarbons have been recognised as the most important occupational lung carcinogens.^6^

Lung cancer accounted for approximate 46 and 44% of all cancers among silicotic workers in a historical cohort of Hong Kong (1981–1999) and Australia (1968–2000).^7,8^ A meta-analysis by pooling the cohort studies of silicosis and lung cancer published after 1997 demonstrated a combined standardised mortality ratio (SMR) of 2.45 (95% CI: 1.63–3.66), but it reduced greatly to 1.60 (95% CI: 1.33–1.93) after smoking was indirectly adjusted.^9^ These statistics indicated that tobacco smoking has contributed to the increased risk of lung cancer among silicotic workers, given the fact that most of silicotics were ever smokers.^7,10^ Tobacco smoking by far is the most important risk factor for lung cancer, contributing to an approximate 80% of lung cancer cases in the European male population^10^ and 58% in Chinese men.^11^ Population-based studies in Asian revealed a sharp decrease of lung cancer risk for over 50% in the first 5–6 years of smoking cessation.^12,13^ A phenomenon of “quitting ill effect (i.e., a higher risk of lung cancer after quitting smoking in a relatively short period of time due to the clinical presentation of illness)” within the first 2 years of smoking cessation was observed in different general populations including Hong Kong.^12,14–16^ Undoubtedly, quitting smoking has beneficial effect on reducing lung cancer risk, but the magnitude of risk reduction may differ between the general population and workers occupationally exposed to silica dust, as the latter group is considered to be at a higher risk of lung cancer due to additionally exposed to occupational carcinogens. Therefore, findings from the general population-based studies may not be directly generalised to the occupational population of workers with silicosis.

Previous occupational epidemiological studies were focused on the carcinogenicity of silica or silicosis by direct or indirect adjustment for tobacco smoking, whereas none of them specifically measure the effect of smoking cessation on lung cancer risk. Until now, it is unclear whether quitting smoking and maintaining the abstinent status also has substantial benefits among workers with silicosis. Moreover, whether silicotic workers have a similar time window of “quitting ill effect” to that of the general population is not known. We used data from a large historical cohort of silicotic workers in Hong Kong to evaluate the impact of smoking cessation on lung cancer mortality, and quantify the exposure-response relationship with years since quitting.

## Methods

### Cohort enumeration and follow-up

During the period 01/01/1981–31/12/2005, we recruited a total of 3202 male workers with silicosis from the Pneumoconiosis Clinic of the Tuberculosis and Chest Service of the Department of Health, and followed up them till 12/31/2014 to determine the vital status and cause of death verified from medical records, with a follow-up rate of 99.5%. All these patients attended Pneumoconiosis Clinic was for the purpose of seeking for compensation rather than routine surveillance. The main reasons for attending Pneumoconiosis Clinic were because of symptoms (41.4%) or referred by the government hospital/clinic (36.5%), while there was a small proportion of patients who were asymptomatic attended the Clinic for check-up (11.3%). The International Classification of Disease (ICD, the 9th Revision) was used to code the underlying causes of death, and the ICD-9th code of lung cancer is 162. A medical panel made the diagnosis of silicosis (profusion category 1/0 or higher) following the criteria of the International Labor Organization^17^ and compensation was entitled regardless of lung cancer impairment. Based on consensus between the two readers, all confirmed silicosis were further classified as large opacities if there appeared any opacity in chest radiographs having a greatest diameter exceeding about 10 mm; otherwise, small opacities were categorised.^17^ The history of pulmonary tuberculosis of each worker was extracted from medical records and each episode of tuberculosis was confirmed by the lab reports of culture and/or sputum smear. We obtained the ethics approval from the Survey and Behavior Research Ethics Committee of the Chinese University of Hong Kong and anonymised the patient records prior to data analysis.

### Exposure assessment on silica dust

We adopted a standardised form to extract each patient’s baseline information from Pneumoconiosis Clinic on age at diagnosis of silicosis, sex, education attainment, place of birth, smoking habit, complete occupational history, and medical history. Participants of this cohort was categorised into three major types of occupation [(i.e., surface construction workers (exposed to silica dust but without other known occupational carcinogens), underground caisson workers (exposed to relatively higher silica dust with evidence of concomitant occupational exposure to radon by direct measurement) and other dusty occupations (exposed to silica dust and other possible occupational carcinogens, e.g., diesel exhaust gases)],^18–20^ and the definitions of these major occupations have been described elsewhere.^7^ We estimated cumulative dust exposure to respirable crystalline silica based on each worker’s complete occupational history in combination with the documented occupational hygiene data on crystalline silica according to a master list of industries and job tasks.^21^ Briefly, a master list of industries and job tasks were constructed using information from each worker’s complete occupational history. We obtained published local occupational hygiene data on respirable crystalline silica dust for the most common job tasks including unskilled laboring, stone cutting, stone crushing machine attendance, pneumatic drilling, underground caisson, and tunneling. A geometric mean of concentration of respirable crystalline silica dust was assigned to each industry or job task for individual workers by a panel of experts including a senior occupational hygienist who was experienced in exposure assessment on silica dust.^21^ Cumulative dust exposure (unit: mg/m^3^-years) was calculated by summation of the products of the concentration of respirable silica dust and the net years of dust exposure from all episodes of job, assuming a constant exposure level over calendar years due to the limited occupational hygiene data being obtained.^7^

### Exposure assessment on tobacco smoking at baseline

Detailed information on smoking habits at the baseline were obtained from the medical records, including status of cigarette smoking, average cigarettes smoked per day, years of smoking, and the year at stopping smoking. An ever smoker was defined as the one who had ever smoked more than 20 packs of cigarettes or 1 cigarette a day for at least 1 year,^22^ otherwise, the worker was a never smoker. A current smoker was further defined if the ever smoker claimed as “I currently smoke”, while a former smoker was defined if he answered “I quit smoking” and then years since quitting were recorded. Smoking pack-years were calculated by multiplying the packs (1 pack = 20 cigarettes) consumed per day and the years of smoking.

### Reassessment of smoking status during the follow-up

Information on smoking habits of each silicotic worker was reassessed each time when he received medical consultation at the Pneumoconiosis Clinic during the follow-up period, normally on a basis of an interval of every 2 years. Five major smoking subgroups were categorised according to the combined information on smoking habits from the baseline and the last reassessment during the follow-up period: (i) never smokers (i.e., never smokers at baseline and throughout the follow-up period); (ii) new quitters (i.e., current smokers at baseline but had quit smoking during the follow-up period); (iii) persistent quitters (i.e., former smokers at the baseline and maintained quitting status throughout the follow-up period); (iv) never quitters (i.e., current smokers at baseline and throughout the follow-up period); (v) new smokers (i.e., never smokers or former smoker at the baseline but started to smoke or re-smoke during the follow-up period). As only 7 patients were new smokers, meaningful analyses were therefore focused on other four major reassessed smoking subgroups of i to iv.

### Statistical analysis

Descriptive analysis was used to compare various characteristics amongst never, former, and current smokers at the baseline. Multiple Cox proportional hazards model was performed to calculate the hazard ratio (HR) and 95% confidence interval (95% CI) of four major smoking subgroups after adjustment of potential confounding factors including age at entry, history of tuberculosis, and cumulative dust exposure. We did not include age at first exposure to silica dust into the model because this variable had no association with lung cancer mortality (OR = 1.00, 95%CI: 0.98–1.02, obtained from univariate regression analysis), and also it had a very weak correlation with cumulative dust exposure (correlation coefficient = 0.09, 95%CI: 0.05–0.12). The proportionality hypothesis was evaluated before performing the Cox proportional hazard models. We examined the exposure-response relationship with years since smoking cessation in four categories (1–10.9, 11–19.9, 20–29.9, ≥30 years) by trend test. In additional, we performed multiple Cox proportional hazards model incorporating the natural cubic spline to model non-linear effects of years since smoking cessation on lung cancer mortality after further adjustment of smoking pack-years, while sensitivity analysis was further carried out after considering the time varying covariate of changing smoking status during follow-up. Analyses were carried out in the R 3.2.3 after loading the “survival”, “Hmisc”, “rms”, “splines” packages.^23^ The minimal Akaike’s information criterion value (a measure of the goodness of fitting a statistical model) was used as a guide to select the best model by fitting various degrees of freedom.^12^ In subgroup analysis, we repeated same multiple Cox proportional hazards model to examine the association between smoking cessation and lung cancer mortality in different subgroups categorised by occupation, radiological severity of silicosis, and history of tuberculosis. As only 17 subjects were loss to follow-up, this report was focused on 3185 silicotics who had complete data on vital status.

## Results

### Patients and baseline characteristics

Among 3185 silicotics enrolled at the baseline, 1505 (47.25%) of them were current smokers and 1353 (42.48%) were former smokers. As shown in Table 1, current smokers had the youngest age at the diagnosis of silicosis and died earlier, and they were more likely to be hired in the underground caisson work and had the highest levels of cumulative silica dust exposure. Compared with the current smokers, former smokers were at a relatively elder age of first exposure to silica dust, more prone to having tuberculosis infection and presenting large opacities in chest radiographs; however, their consumptions of smoking pack-years did not differ significantly from those of the current smokers.

**Table 1:** Characteristics of 3185 silicotic workers in subgroups of smoking status at baseline

Characteristics	All subjects	Smoking status			*P* value
		Never smokers	Current smokers	Former smokers	
No. of subjects	3185	327 (100.00)	1505 (100.00)	1353 (100.00)	
No. of lung cancer deaths (all deaths)	188 (1942)	4 (168)	122 (975)	62 (799)	
Age at entry	55.28 ± 10.51	54.80 ± 10.83	53.36 ± 9.80	57.54 ± 10.76	<0.001
Age at death	68.07 ± 10.70	69.11 ± 11.56	67.19 ± 10.58	68.92 ± 10.59	0.001
Smoking pack-years	25.72 ± 23.24	0	28.33 ± 20.63	29.05 ± 24.92	0.426
History of tuberculosis^a^					
Yes	1552(48.82)	149(45.71)	651(41.95)	752(55.70)	<0.001
No	1627(51.18)	177(54.29)	852(52.37)	598(44.30)	
Place of birth^b^					
Hong Kong	183(5.76)	38(11.66)	80(5.33)	65(4.80)	<0.001
Mainland	2948(92.73)	283(86.81)	1400(93.33)	1265(93.50)	
Others	48(1.51)	5(1.53)	20(1.33)	23(1.70)	
Age at first exposure to silica (years)	24.70 ± 7.31	23.47 ± 7.46	24.57 ± 7.21	25.14 ± 7.35	0.001
Cumulative dust exposure (mg/m^3^-year)	449.87 (130.00- 590.12)	410.00 (79.17- 588.75)	461.84 (150.88 - 587.34)	432.38 (100.89- 603.67)	0.018
Years of silica dust exposure	24.67 ± 9.57	25.16 ± 9.92	24.43 ± 9.22	24.83 ± 9.87	0.340
Occupational groups					
Surface construction	1619(50.83)	178(54.43)	738(49.04)	703(51.96)	0.044
Underground caisson	1204(37.80)	103(31.50)	600(39.87)	501(37.03)	
Others	362(11.37)	46(14.07)	167(11.10)	149(11.01)	
Radiological severity of silicosis					
Small opacities only	2542 (79.81)	259 (79.20)	1261 (83.79)	1022 (75.54)	<0.001
Large opacities	643(20.19)	68 (20.80)	244 (16.21)	331(24.46)	

*Note*: Values are given as *n* (%) for categorical variables, mean ± SD for normal distributed continuous variables and median (Q1-Q3) for non-normal distributed continuous variables. The differences between the proportions were tested by Chi-square test, and the mean differences were tested by ANOVA between the subgroups. Further, Mann–Whitney *U* test was carried out for the non-normal distribution data

^a^Excluding 6 workers with unknown history of tuberculosis

^b^Excluding 6 workers with unknown place of birth

### Exposure-response relationship with years since cessation

**Fig. 1:**
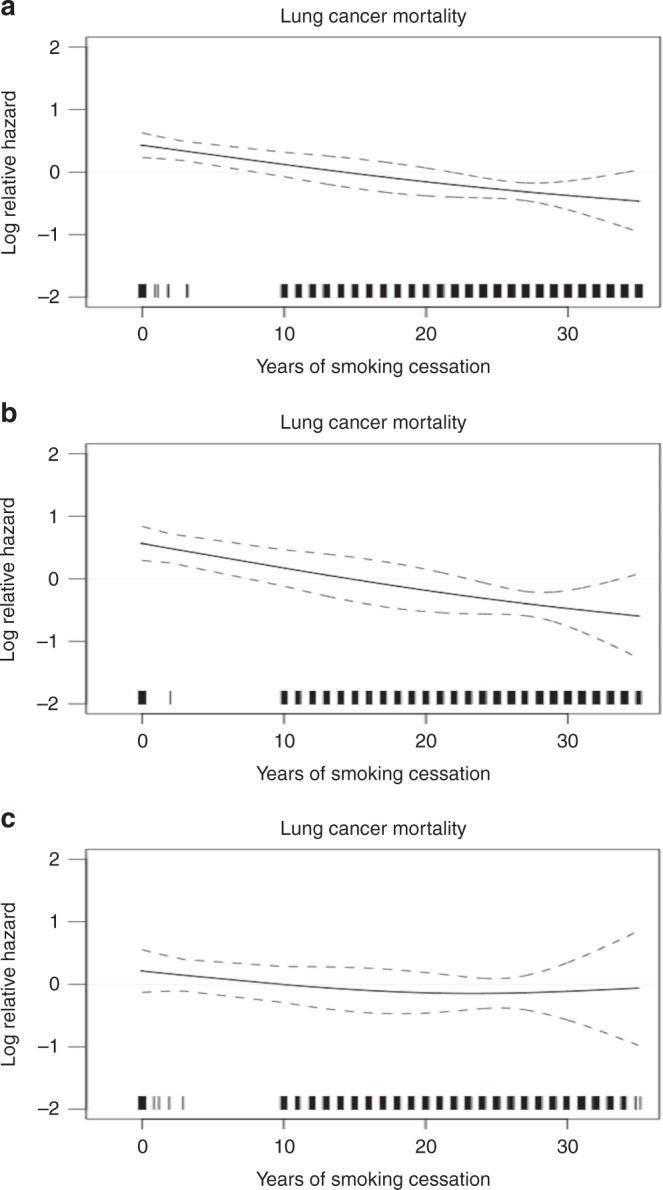
Exposure-response relationships between years since smoking cessation and lung cancer mortality among (**a**) all silicotic workers, (**b**) surface construction workers, (**c**) underground caisson workers. The curves were plotted by multiple Cox proportional hazards model incorporating nature cubic spline curve with degree of freedom of three

Table 2 shows the effects of years of smoking cessation on lung cancer mortality among silicotics. Compared with the never quitters, a significantly negative gradient (*p* *=* 0.001 for trend test) of lung cancer mortality was observed with increasing years since smoking cessation for all silicotics, despite the significant risk reduction did not manifest in the first 10 years of cessation. Overall, the risk of lung cancer mortality among all silicotics was nearly halving within 20 years since cessation (HR = 0.54, 95%CI: 0.35–0.83), and it further decreased by 68% (HR = 0.32, 95%CI: 0.48–0.81) if the smokers continued to abstain from cigarette smoking for 30 years or more. The risk reduction for surface construction was even more evident in the first 20 years of cessation (HR = 0.44, 95%CI: 0.24–0.83) but it showed a similar decease by 68% in the subsequent years; nevertheless, the risk could not revert back to that of never smokers. Fig. 1 depicts the dynamic risk of lung cancer mortality with increasing years of smoking cessation using smoothing spline analysis, based on a continuous scale to cover a full range of years since cessation. An almost linear trend of the decreased HR was indicated with increasing years since cessation for all silicotics (Fig. 1a) and surface construction workers (Fig. 1b), but the long-term beneficial effect was weakened for the underground caisson workers (Fig. 1c). Results from sensitivity analysis by further considering changes of smoking status during follow-up into the model did not show obvious variations in the effects since smoking cessation (Supplement Fig. 1).

**Table 2:** Hazard ratio (HR, 95% confidence interval) of lung cancer mortality according to years since smoking cessation among silicotic workers in different types of occupation ^a^

Levels of exposure	Types of occupation		
	Entire cohort	Surface construction	Underground caisson
Years since cessation			
Never quitters	1.00 [87]	1.00 [53]	1.00 [23]
1–9.99	0.72 (0.29–1.79) [5]	0.46 (0.06–3.25) [1]	1.01 (0.34–2.97) [4]
10–19.99	0.54 (0.35–0.83) [28]	0.44 (0.24–0.83) [13]	0.85 (0.44–1.63) [15]
20–29.99	0.55 (0.38–0.80) [43]	0.47 (0.28–0.78) [21]	0.63 (0.32–1.23) [14]
≥30	0.32 (0.19–0.52) [20]	0.32 (0.17–0.59) [13]	0.46 (0.17–1.22) [6]
Never smokers	0.11 (0.04–0.30) [4]	0.08 (0.02–0.36) [2]	0.28 (0.06–1.22) [2]

^a^Multiple Cox proportional hazards models were adjusted for age at entry, place of birth, history of tuberculosis, smoking pack-years, and cumulative exposure of silica

[ ]: no. of lung cancer deaths

### Effects of reassessed major smoking groups during follow-up

As of 31 December 2014, 1942 deaths occurred and 188 lung cancer deaths were identified.

A total of 643 silicotics (16.9%) were current smokers at baseline but quitted smoking during the follow-up (i.e., new quitters). There were 934 (24.4%) silicotics who were active smokers at baseline and kept smoking status follow up (i.e., never quitters). A majority of former smokers at baseline (1279/1353, 94.5%) had sustained their quitting status over the entire study period. Compared with the never quitters (Table 3), persistent quitters (i.e., former smokers at baseline and kept abstinence of smoking during follow-up) had a 53% decrease in lung cancer mortality (adjusted HR = 0.47, 95%CI: 0.33–0.66). New quitters had about half the risk of never quitters (adjusted HR = 0.51, 95%CI: 0.34–0.76), whilst the HR for never smokers was only 0.11 (95%CI: 0.04–0.31). Similar patterns of risk were also observed among surface construction workers and those with small opacities. However, the results tended to be less stable for the underground caisson workers and those with large opacities due to a small number of cases. More notable decrease in lung cancer mortality was seen among silicotics with a history of tuberculosis than their counterparts for either the persistent or new quitters.

**Table 3:** Hazard ratios (HR, 95% confidence interval) of lung cancer mortality for the major groups of smoking cessation reassessed during the follow-up period (1981–2014) according to subgroups categorised by types of occupation, radiological severity of silicosis at diagnosis, and history of tuberculosis

	Major smoking groups			
Characters of exposure	Never smokers	Persistent quitters	New quitters	Never quitters
Entire cohort^a^	0.11(0.04–0.31) [4]	0.47(0.33–0.66) [61]	0.51(0.34–0.76) [35]	1.00 [87]
Surface construction	0.09(0.02–0.36) [2]	0.41(0.26–0.64) [33]	0.42(0.24–0.74) [15]	1.00 [53]
Underground caisson	0.28(0.06–1.24) [2]	0.67(0.37–1.22) [22]	0.73(0.39–1.37) [17]	1.00 [23]
Radiological severity of silicosis^a^				
Small opacities	0.10(0.03–0.32) [3]	0.38(0.26-0.57) [41]	0.52(0.35–0.78) [33]	1.00 [77]
Large opacities	0.20(0.02–1.67) [1]	1.08(0.49-2.38) [20]	0.31(0.07–1.43) [2]	1.00 [10]
History of tuberculosis^b^				
No	0.08(0.02–0.32) [2]	0.50(0.32–0.78) [34]	0.59(0.36–0.95) [25]	1.00 [53]
Yes	0.16(0.04–0.70) [2]	0.42(0.25–0.69) [27]	0.39(0.19–0.80) [10]	1.00 [34]

^a^Adjusted by age at entry, place of birth, history of tuberculosis, smoking pack-years, and cumulative exposure of silica

^b^Adjusted by age at entry, place of birth, smoking pack-years, and cumulative exposure of silica

[ ]: no. of lung cancer deaths

## Discussion

This historical cohort study over 30 years of follow-up is the first to demonstrate that quitting smoking and maintaining the abstinent status was associated with a substantial reduction in lung cancer mortality among silicotic workers. Importantly, an almost linear decrease in trend of lung cancer mortality was observed with increasing years since smoking cessation, despite the significant benefit was only manifest after 10 years of abstinence and the long-term beneficial effect was attenuated for underground caisson workers. Overall, lung cancer mortality nearly halved in the first 20 years of quitting, and continued to decrease in the subsequent years, with an overall reduced risk of 68% both for all silicotics  and surface construction workers, while the risk reduction was 54% for underground caisson workers.

Existing evidence left little doubt that quitting smoking has beneficial effect of reducing lung cancer risk,^24,25^ but the extent of risk reduction varied amongst general populations ranging from 1.3 (95%CI: 1.2–1.5) in US to 9.35 (7.05–12.4) in Canada, as shown in a meta-analysis report which included 8 case-control studies with all types of lung cancer cases.^24^ Findings from the Singapore Chinese Health Study among 45,900 participants demonstrated a 28% risk reduction of lung cancer (adjusted HR = 0.72, 95%CI: 0.53–0.98) for the new quitters and a 58% risk reduction (adjusted HR = 0.42, 95%CI: 0.32–0.56) for the continuous ex-smokers; however, the relatively short follow-up of 6 years precluded a more detailed exposure-response analysis with smoking cessation.^13^ A population-based case-control study among Hong Kong males (1208 cases, 1089 controls) addressed this knowledge gap and reported a rapidly decreasing lung cancer risk by 54% in the first 2–5 years of quitting, with slower rate of decrease in the subsequent years.^12^ Globally, most epidemiological studies regarding smoking cessation and lung cancer risk were conducted in general populations, but this topic has been rarely investigated in occupational epidemiological studies among workers with silicosis.

We systematically searched literature from Medline and PubMed since 1966 and obtained only 5 epidemiological studies (Canada, US, Japanese, and 2 Finnish studies) examining the effect of former smoking (versus never smokers) on lung cancer risk among silicotic workers, with a risk estimate ranging from 1.90 to 3.52.^26–30^ Compared with the former smoking silicotics, RR of lung cancer mortality was 1.68 (95%CI: 1.02–2.79) among current smoking silicotics and the corresponding RR was 2.53 (95%CI: 1.41–4.54) for the incidence data,^30^ indicating a decreased lung cancer risk among workers with silicosis who stopped smoking. We assessed the methodological quality of these 5 pertinent studies using Downs and Black checklist^31^ and the quality was moderate. Specifically, these 5 pertinent studies were relatively small and only presented age-adjusted effect measure, while they did not provide detailed information on types of cessation (e.g., persistent quitter, new quitter, never quitter) and years of cessation. Moreover, these previous studies did not well define smoking status.

Population-based epidemiological studies in Hong Kong and other countries pointed out that the benefit from smoking cessation on reducing lung cancer risk was not immediately initiated from the time of giving up smoking, but demonstrated an accelerated risk reduction after a 2-year window since the start of smoking cessation “quitting ill effect”,^12,14–16^ and this might be interpreted as smokers choosing to quit smoking because of early symptoms before diagnosis of lung cancer. While significant benefit among our silicotics only appeared after 10 years of abstinence, there was no clear cutoff time window of quitting ill effect. Compared with the general population, workers with silicosis have irreversible lung impairment and it may progress even if without further exposure to silica dust or smoking. Consequently, workers with silicosis, particularly for those ever hired in underground caisson, may require a longer time to manifest the beneficial effect from smoking cessation than the general population who are supposed to have relatively healthy lungs. Silicotics in underground caisson were more susceptible than the surface construction workers, as more of the caisson workers presented large opacities and had concomitant exposure to radon daughter in addition to a higher level of silica dust exposure.^7^

The most important feature of this large occupational historical cohort study is the use of repeated measurements on smoking status during the follow-up period from 1981 to 2014. Although smoking and cessation status were not verified by measuring markers (e.g., urinary cotinine), information on smoking status of each silicotic was strictly reassessed by trained occupational nurses for each medical visit at Pneumoconiosis Clinic. Assessment of smoking behaviors among our silicotics, however, may not be in a same interval between each visit, and thus may introduce non-differential misclassification bias to our study, which in turn, may have led to an underestimation of the true risk estimate. We performed subgroup analyses according to occupation, radiological severity of silicosis and history of tuberculosis, and therefore a potential Type I error may also be a concern because of these extra subgroup analysis. We did not collect other confounding factors (e.g., family cancer history) which may introduce confounding bias to our study; however, this uncontrolled confounder may lead to an underestimation of the effect of interest, as the distribution of the potential confounder was likely to be randomly distributed among smoking subgroups. Differences in diagnostic technologies for lung cancer over time may be a concern as the diagnostic technology for lung cancer has improved largely during the last 30 years, which is reflected in the report of Hong Kong Cancer Registry that there is an annual reduction rate of 9.25% for lung cancer cases without histological confirmation^11^; however, they are likely to introduce non-differential misclassification leading to a null association.

## Conclusions

Our study demonstrated that smoking cessation has substantial benefit to reduce lung cancer mortality among silicotics, and further provides new evidence that new quitters of smoking had halved their risks of dying from lung cancer, while the benefit was greater among the persistent quitters. Given very high prevalence of smoking among silicotics, findings from our study underscore the importance in occupational health practice to prevent workers from smoking and develop effective control measures in helping particularly the obstinate smokers quit smoking.

## Electronic supplementary material

Supplemental Figure 1A

Supplemental Figure 1B

Supplemental Figure 1C

## Availability of data and material

We do not open our data to the public, but the readers can approach the corresponding author for some limited usage of the data.
