# Supplementary figures and images for: Smoking cessation sharply reduced lung cancer mortality in a historical cohort of 3185 Chinese silicotic workers from 1981 to 2014
Source: Br J Cancer. 2018 Nov 13;119(12):1557–62. doi: 10.1038/s41416-018-0292-6 (PMC6288151; doi:10.1038/s41416-018-0292-6)

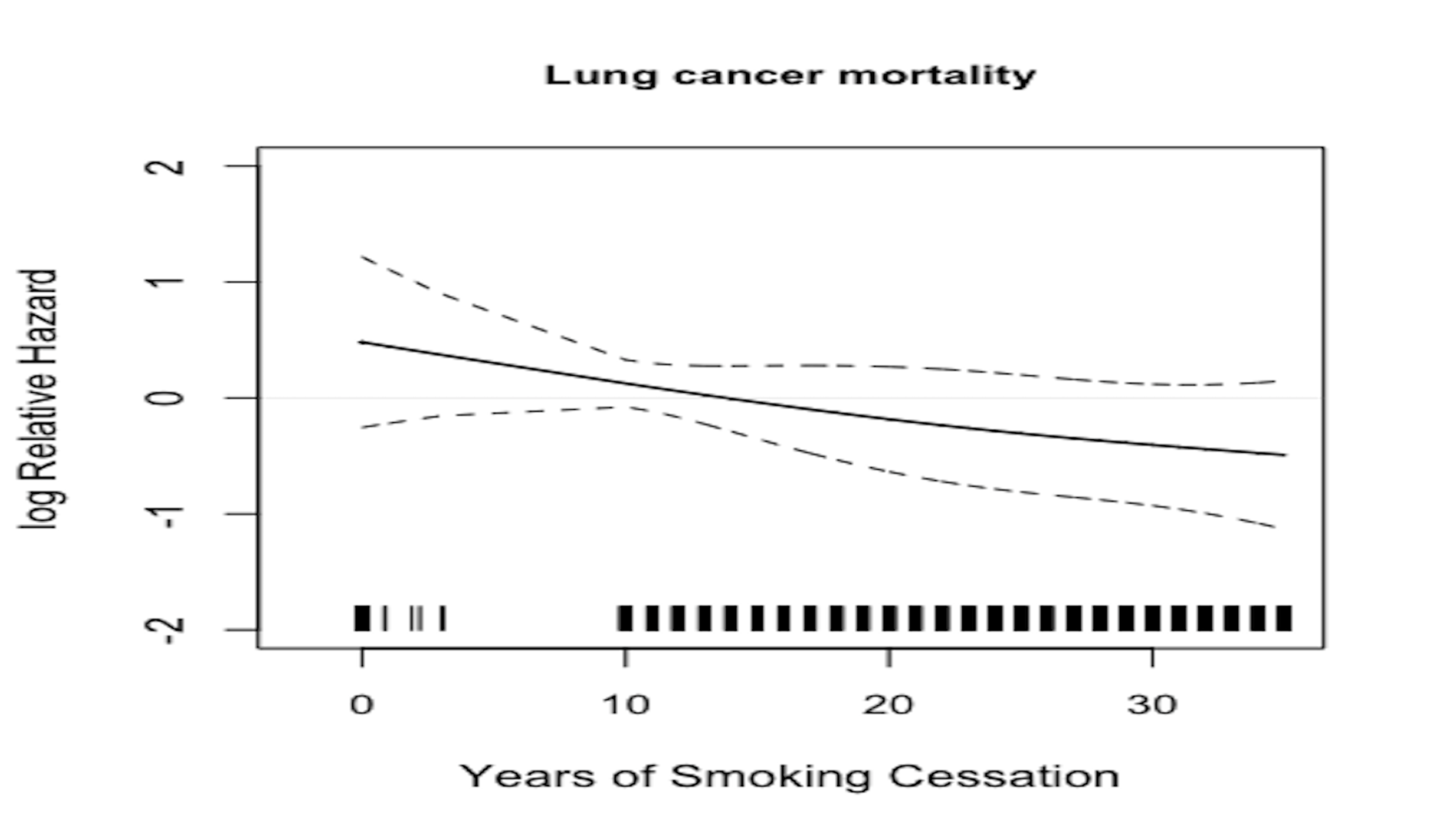

Supplement: Supplementary file 1 — Supplemental Figure 1A [file 41416_2018_292_MOESM1_ESM.png]

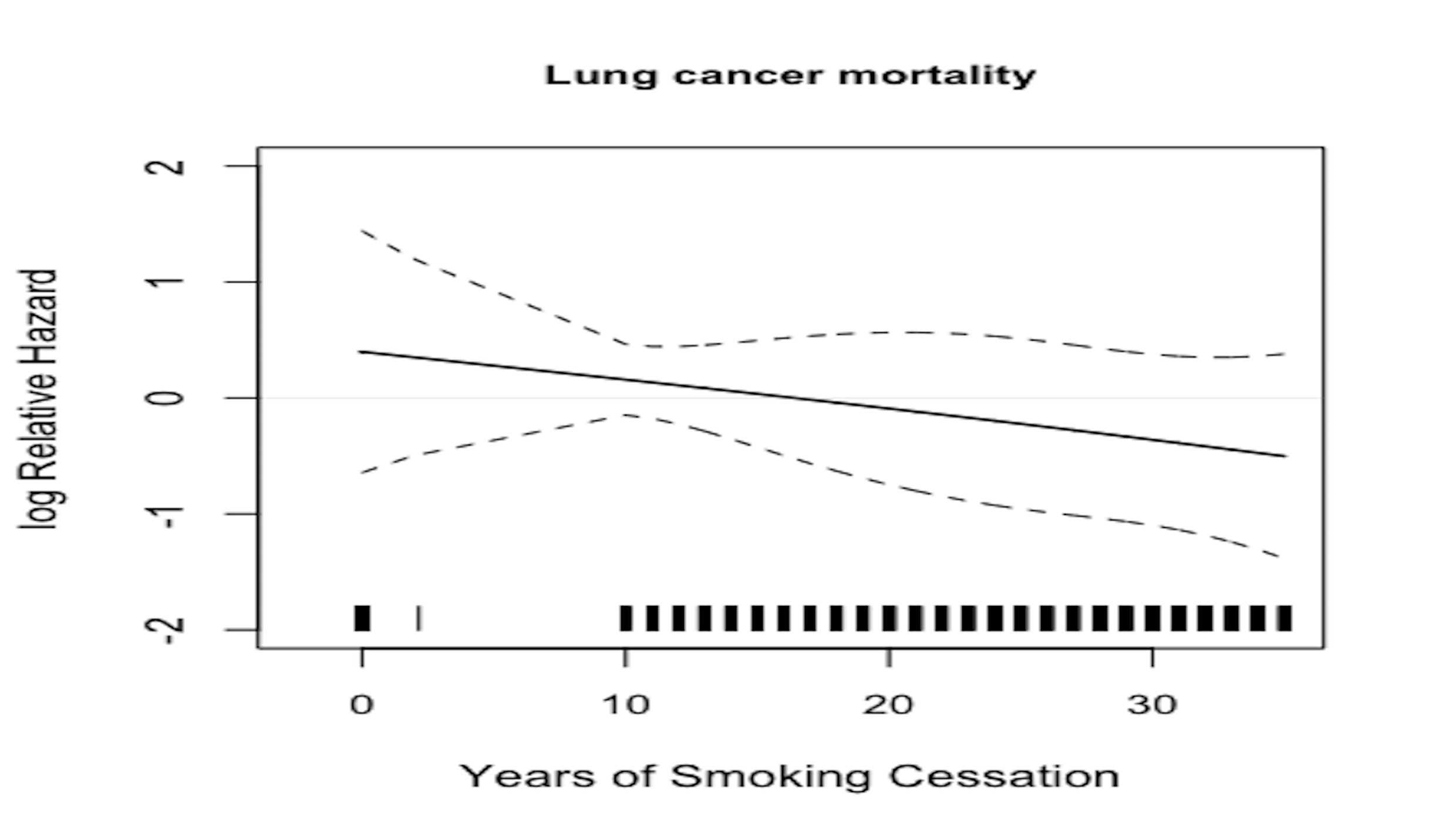

Supplement: Supplementary file 2 — Supplemental Figure 1B [file 41416_2018_292_MOESM2_ESM.png]

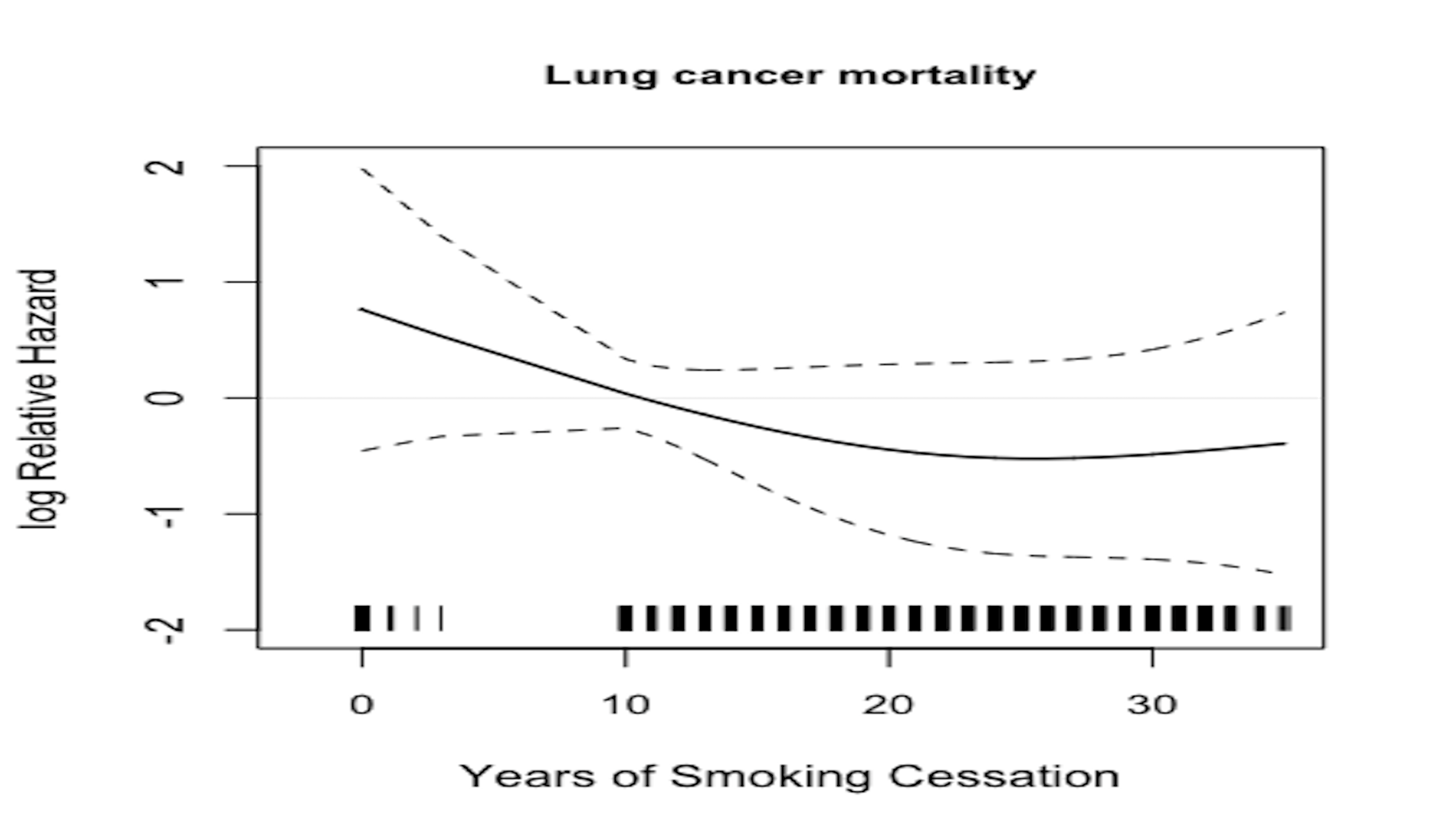

Supplement: Supplementary file 3 — Supplemental Figure 1C [file 41416_2018_292_MOESM3_ESM.png]
